# miR-378-3p maintains the size of mouse primordial follicle pool by regulating cell autophagy and apoptosis

**DOI:** 10.1038/s41419-020-02965-1

**Published:** 2020-09-10

**Authors:** Xiaowen Sun, Francesca Gioia Klinger, Jing Liu, Massimo De Felici, Wei Shen, Xiaofeng Sun

**Affiliations:** 1 College of Animal Science and Technology, Qingdao Agricultural University, Qingdao, 266109 China; 2 College of Life Sciences, Qingdao Agricultural University, Qingdao, 266109 China; 3 Department of Biomedicine and Prevention, University of Rome Tor Vergata, Rome, 00133 Italy; 4 Central laboratory of Qingdao Agricultural University, Qingdao, 266109 China

**Keywords:** Mitophagy, miRNAs, Infertility

## Abstract

Primordial follicle pool provides all available oocytes throughout the whole reproductive life span. Abnormal regulation in primordial follicle assembly leads to abnormal size of primordial follicle pool, even causes infertility. Here, miR-378-3p was proved to regulate mouse primordial follicle assembly both in vivo and in vitro. The expression of miR-378-3p significantly increased in mice ovaries from 17.5 dpc (days post coitum) up to 3 dpp (day post partum) compared with the expression of 16.5 dpc ovaries, which suggested that miR-378-3p was involved in primordial follicle assembly. To uncover the underlying mechanism, newborn mice ovaries were cultured in vitro in the presence of rapamycin and 3-methyladenine, which showed that the expression of miR-378-3p changed together with the percentage of primordial follicle. Moreover, during the normal process of primordial follicle assembly between 17.6 dpc and 3 dpp, autophagy is activated, while, apoptosis is inhibited. The in vivo results showed that newborn mice starved for 1.5 days showing the increased miR-378-3p, activated autophagy and inhibited apoptosis in the ovaries, had more percentage of primordial follicles. Over-expression of miR-378-3p using miR-378-3p agomir caused increased percentage of primordial follicle, increased level of autophagy, and decreased level of apoptosis. Knockdown of miR-378-3p by miR-378-3p antiagomir had the opposite results. Using pmirGLO Dual-Luciferase miRNA Target Expression system, we confirmed both PDK1 and Caspase9 were targets of miR-378-3p, which suggested that miR-378-3p activated autophagy by targeting PDK1 and inhibited apoptosis by targeting Caspase9. MiR-378-3p could be used as a biomarker of diseases caused by abnormal size of primordial follicle pool for diagnosis, prevention, or therapy.

## Introduction

In mammals, the establishment of primordial follicle pool is an important process, which provides all available oocytes throughout the whole reproductive life span^1–3^. Germ cells, after migrating to the genital ridges, undergo mitosis with incomplete cytokinesis, therefore closely associated clusters of syncytic germ cells are gathered to form germ cell cyst^4,5^. During primordial follicle assembly around birth (17.5 dpc–5 dpp in mice), pre-granulosa cells migrate into the cyst, leading to their breakdown, and surround the oocyte forming primordial follicle, in which single oocytes are enclosed by a layer of flattened pre-granulosa cells^6^. The process of primordial follicle assembly is vital for the size of primordial follicle pool. But the abnormal primordial follicle assembly leads to diseases of female reproductive system even causes infertility^7^. During the process of germ cell cyst breakdown and primordial follicle assembly, approximately two-thirds of germ cells undergo loss before or shortly after birth in mice^8,9^. Most previous studies reported that programmed cell death also named cell apoptosis accounted for the germ cell loss during this process^8,10,11^. Also autophagy is reported to be involved in primordial follicle assembly. In fact, autophagy is confirmed to be active in mice perinatal ovary to prevent germ cell over-loss^12,13^, probably because active autophagy can accumulate nutrient substances to better supply the surviving oocyte^14^. It is well known that autophagy can be induced by starvation^15–17^. In mammals, the fetuses get their nutrition from the mother through placenta, and after birth, they get their nutrition from the milk. After parturition and before sucking milk, newborns experience a period of several hours of starvation. This starvation is enough to start autophagy to regulate cell number and energy equilibrium. Among all the reports, the role of autophagy in primordial follicle assembly can be the pro-death signaling leading to the loss of germ cells or can be the pro-survival signaling to protect the germ cells in ovaries from over-loss. Autophagy is an evolutionarily conserved process from yeast to mammals, during which double membraned autophagosomes containing cytoplasm and intracellular organelles are formed. Microtubule-associated protein 1 light chain 3β (LC3B) plays an important role in autophagy. During autophagy, the cytosolic from of LC3B-I, transforms into LC3B-II that binds to the membrane of autophagosomes and induces punctuate structures^18^. Therefore LC3II/LC3-I can be regard as the marker of autophagy and the punctate structure can determine the degree of autophagy. Many reports have suggested that the regulation of autophagy may be through the cross talk between autophagy and apoptosis, because the autophagy associated protein Beclin1 has BH3 region, which is a binding site for B-cell lymphoma/leukemia-2 (BCL-2)^18^. The binding of BCL-2 with Beclin1 affects autophagy, and the binding of BCL-2 with Bax/Bak affects apoptosis^19,20^. However, during germ cell cyst breakdown and primordial follicle assembly, the exact effects of autophagy and apoptosis in germ cell loss and the underlying mechanism are unclear. Uncovering the regulating mechanism is helpful to seek the biomarkers and potential targets for early diagnosis, prevention, and therapy of the related diseases.

MiR-378 is a vital epigenetic regulator in the mammalian ovaries, whose expression changes with the stages of the ovarian development. Pan et al. reported that MII cumulus cells had a significant lower expression level of miR-378 than that in germinal vesicle (GV) cumulus cells in porcine cumulus-oocyte complexes (COCs) during in vitro culture, indicating a role of miR-378 in oocyte maturation^21^. Our previous study also discovered the regulating role of miR-378 in oocyte maturation and follicle development using in vivo animal model^22^. Moreover miR-378 has been recently proposed to be a critical component of metabolic checkpoints, enhancing the autophagic process and blocking apoptotic initiation in other cell types^23^.

In this study, we discovered that germ cells in the ovary of newborn mice underwent autophagy (within 1.5 days starvation) and then apoptosis (beyond 2 days starvation) after nutrition deprivation. During the whole process, miR-378 played regulating roles by targeting phosphoinositide-dependent protein kinase 1 (PDK 1), mammalian target of rapamycin (mTOR), and caspases, which suggested that miR-378-3p could be further used as a biomarker for abnormal size of primordial follicle pool and a potential therapeutic target for diseases caused by abnormal primordial follicle assembly.

## Materials and methods

### Ethics statement

All the animal procedures were examined and approved by the Ethics Committee of Qingdao Agricultural University (approval no. 2019012).

### Animals

CD1 mice used in the project were purchased from Vital River Laboratory Animal Technology Co. LTD (Beijing, China) and maintained in the animal house of Qingdao Agriculture University, under condition of controlled light (12 h for light, 12 h for dark, cycle) and temperature (21–22 °C) with free access to food and water. Totally, 210 female mice were mated and the vaginal plugs could be observed the next morning (considered as 0.5 dpc, days post coitum) from 73 mice. Among them, 34 were used for in vivo experiment. Ovaries used in this experiment were collected from mice of 16.5 dpc, 17.5 dpc, 0 dpp (days post partum), 1.5 dpp, 2 dpp and 3 dpp (4 pregnant mice per stage), which were euthanized by cervical dislocation. Newborn female mice (from 10 mother) were randomly divided into three groups, one group had normal milk breastfeeding, while the other two groups were separated from their mothers immediately after birth (within 6 h) to induce starvation by deprivation of milk supply for 1.5 days (1.5 dn group) or 2 days (2 dn group), respectively. Two days starvation is the survival limiting time point since a small number of pups died at this time point. Only survivors were sacrificed to collect the ovaries.

### In vitro culture of ovaries

Ovaries of newborn mice (0 dpp) were collected and cultured in wells of 24-well plates under the condition of 37 °C with 1 mM 3-Methyladenine (3-MA, autophagy inhibitor, HY-19312, MedChemExpress), 100 μM Rapamycin (Rap, autophagy activator, HY-10219, MedChemExpress) and DMSO as control for 3 days. The medium used were DMEM/F12 (HyClone, SH30023.01B, Beijing, China) and a-MEM (HyClone, SH30265.01B, Beijing, China) (1:1) supplemented with 10% FBS (Gibco, 10099-141, USA), 100 IU/ml of penicillin G, and 100 mg/ml of streptomycin sulfate (penicillin-streptomycin solution, HyClone, SV30010, Beijing, China). MiR-378-3p agomir and miR-378-3p antiagomir were used for miR-378 over- and down-expression. They were respectively added to the medium at the concentration of 200 nM at the beginning of the culture and the ovaries were then cultured in vitro for 3 days at the conditions described above. MiR-378-3p agomir and miR-378-3p antiagomir were designed and synthesized by Ribobio company (Guangzhou, China). The sequences of miR-378-3p agomir was as following: GGAAGACUGAGGUUCAGGUCA. The miR-378-3p antiagomir was the specifically modified reverse complementary sequences of miR-378-3p agomir. Caspase9 siRNAs and negative control siRNA were designed and synthesized by GenePharma company (Shanghai, China). 50 pM siRNA were used to silence Caspase9. Totally, 39 mother mice were used for in vitro experiment, 12 (4 in control group, 4 in Rap group and 4 in 3-MA group) for Rap/3-MA experiment, 24 (8 in control group, 8 in miR-378-3p agomir group, and 8 in miR-378-3p antiagomir) for miR-378-3p agomir/miR-378-3p antiagomir experiment and 3 for Caspase9 RNA interference.

### Small RNA extraction and miRNA qRT-PCR

Total RNA was extracted by EASYspin Plus RNA extraction kit (RN2802, Aidlab, Beijing, China) according to the instruction manual. Then, miRNAs were reverse transcribed with Mir-X^TM^ miRNA First-Strand Synthesis Kit (1804801 A, TaKaRa). Primers of miR-378-3p-F and miR-378-3p-R used in the experiment were synthesized by TSINGKE biological technology (Beijing, China). TBGreen^TM^ Premix Ex Taq^TM^ II (RR820A, TaKaRa) was used for PCR. The PCR conditions were as follows: 10 min at 95 °C, followed by 45 cycles of 95 °C for 10 s, 60 °C for 30 s and 72 °C for 20 s. The relative transcription amount for each miRNA was calculated using 2-∆∆ (target miRNA CT value - U6 CT value).

### Immunofluorescence staining

Ovaries from different ages (16.5 dpc–3 dpp) and different treatments (starvation treatment, Rap treatment, 3-MA treatment, miR-378 overexpression, and knockdown) were collected for immunofluorescence staining, according to standard protocol. Briefly, after overnight fixation with 4% paraformaldehyde solution, samples were paraffin-embedded and serially sectioned in 5 μm sections. For each ovary, about 30 serial sections were obtained. Antigen retrieval was performed in trisodium citrate at 96 °C for 10 min. After cooling at room temperature, the slides were treated for 45 min with blocking solution BDT (30 mg BSA and 100 μl goat serum dissolved in 900 μl TBS). To quantify germ cells and follicles, the germ cell-specific marker MVH was used^24,25^. The sections were incubated with primary antibodies anti-MVH (ab13840, Abcam), LC3B (Ab51520, Abcam), CASP9 (Ab202068, Abcam), CASP3 (Ab13847, Abcam), and Stat3 (9139p, Cell signaling technology) diluted in blocking solution at 1:150 for 8 h at 4 °C, and they were then incubated with FITC/Cy3 labeled secondary antibodies (1:150, Goat pAb to Mouse IgG, ab 150113, abcam; Goat pAb to Rabbit IgG, ab 181448, abcam; Donkey pAb to Rabbit IgG, ab 150074, abcam) at 37 °C for 40 min. Hoechst33342 (B2261, Sigma, USA) was used to stain the nuclei. Germ cells in follicles or within cysts were distinguished and counted under a fluorescent microscope (Olympus, BX51, Japan). One of four sections was selected for counting germ cells.

### TUNEL staining

To further confirm the apoptotic level of ovarian cells, TUNEL staining was performed using BrightGreen Apoptosis Detection Kit (Vazyme, A112-03, Nanjing, China) following the instructions. After serially sectioned in 5 μm sections, they were treated with proteinase. TUNEL reaction solution was then made by 5:1 Label Solution and Enzyme Solution to incubate the sections for 60 min at 37 °C. The cell nuclei were finally stained with Hoechst33342. BX51 Olympus fluorescence microscope (Olympus, BX51, Japan) was used for imaging the fluorescent signals.

### Western blotting

Ovaries from different treated groups were collected for western blotting analysis according to the standard method^26^. The proteins from each group were homogenized and extracted using RIPA lysis buffer (5 ul buffer for one ovary). Proteins were separated by running SDS page gel (6% separating gels for mTOR and p-mTOR, 12% for other proteins) and transferred to PVDF membranes (wet transfer for mTOR and p-mTOR, semi-dry transfer for other proteins). Non-specific binding was blocked by blocking solution (10% BSA in TBST) at 4 °C for 4 h. Then the membranes were incubated overnight in the primary antibodies at concentration of 1 μg/ml. The primary antibodies are as following: anti-mTOR (A104087094, BiOSS), anti-p-mTOR Ser2448 (D9C2, Cell signaling technology), anti-PDK1 (D155182-0025, Sangon Biotech), anti-LC3B (ab51520, Abcam), anti-ACTIN (D110007, Sangon Biotech), anti-CASP9 (ab202068, Abcam), anti-GAPDH (YM3040, Immunoway), anti-CASP3 (Ab13847, Abcam), anti-BCL-2 (AB112-1, Beyotime biotechnology) and anti-BAX (2772 S, Cell signaling technology). After 3 times washing in TBST, membranes were incubated in HRP-conjugated secondary antibodies (A0216, Goat anti-Mouse IgG, Beyotime, China and A0208, Goat anti-Rabbite IgG, Beyotime, China) for 1.5 h. The bands were visualized by chemiluminescent method using BeyoECL Plus kit (Beyotime, P0018S, Shanghai, China). The intensity of specific bands was digitally quantified using Actin as internal reference with AlphaView SA software.

### Generation of the 3′-UTR expression constructs and expression analysis

The targets of miR-378-3p were predicted by mirTargets software. Among the targets, we chose PDK1, an autophagy-related gene and Casspase9, an apoptosis-related gene for further study. miRWalk (http://mirwalk.umm.uni-heidelberg.de/) was used to predict the candidate miR-378-3p binding sites. The ~200 bp 3′UTR sequences, including the binding sites, were synthesized by TSINGKE biological technology (Beijing, China) and cloned into the pmirGLO Dual-Luciferase miRNA Target Expression Vector. At the same time, the mutations were also synthesized and cloned into the pmirGLO Dual-Luciferase miRNA Target Expression Vector. The detailed sequence information is shown in Fig. S1.

293FT cells were seeded into the wells of a 24-well plate. When the cells reached 60% confluence, transfection was performed with wild 3′UTR and miR-378-3p agomir using Lipofectamine 2000 reagent and 293FT cells transfected with wild 3′UTR only were considered as control. Mutation 3′UTR vector with or without miR-378-3p agomir was also transfected to the 293FT cells. 24 h later, cell lysates were harvested by direct lysis. Luciferase activity was measured in triplicate using the Dual Luciferase Assay System (RG027, Beyotime, China) following the manufacturer’s protocol. Firefly luciferase activity was normalized by renilla luciferase activity. Briefly, the samples were added to the wells of 96-well plate; then 100 μl of firefly luciferase assay reagent was added, and RLU (relative light unit) was determined after mixed thoroughly. Then, the mixed samples were added to 100 μl of Renilla Luciferase Detection Working Solution, and the RLU was determined again. The RLU value was finally obtained using the RLU value of firefly luciferase divided by the RLU value of the renilla luciferase.

### Flow cytometry analysis

Ovaries were collected and digested into single cells (30–45 min) with 2 mg/ml collagenase (C5138, Sigma, USA). The cells were then fixed with 80% ethanol for 5 min. and incubated overnight with MVH primary antibody (1:150, ab13840, abcam), following secondary antibody (1:200, ab181448, abcam) incubation at 37 °C for 1 h. Finally, fluorescence intensity was analyzed by flow cytometry (BD Calibur, CA, USA) with Flowjo 7.6 software.

### Measurement of autophagic flux

Bafilomycin A1 induced LC3B-II accumulation was performed. During the culture of 0 dpp ovaries in vitro with miR-378-3p agomir, miR-378-3p antiagomir and respective control, 100 nM Bafilomycin A1 (Baf-A1, A601116-0025, BBI Life science) was used for induction. Then the treated ovaries were harvested for western blotting, and the amount of LC3B-II/GAPDH was compared.

### Statistical methods

All data were analyzed with GraphPad Prism software and were represented as the mean ± SD of at least three independent experiments. Statistical differences between two groups were analyzed by *T* test. And statistical differences between each groups of more than three groups were analyzed and determined by a one-way analysis of variance (ANOVA) followed by the Tukey’s test. The results were regarded as significance when *P* < 0.05.

## Results

### MiR-378-3p regulates autophagy during primordial follicle assembly

**Fig. 1:**
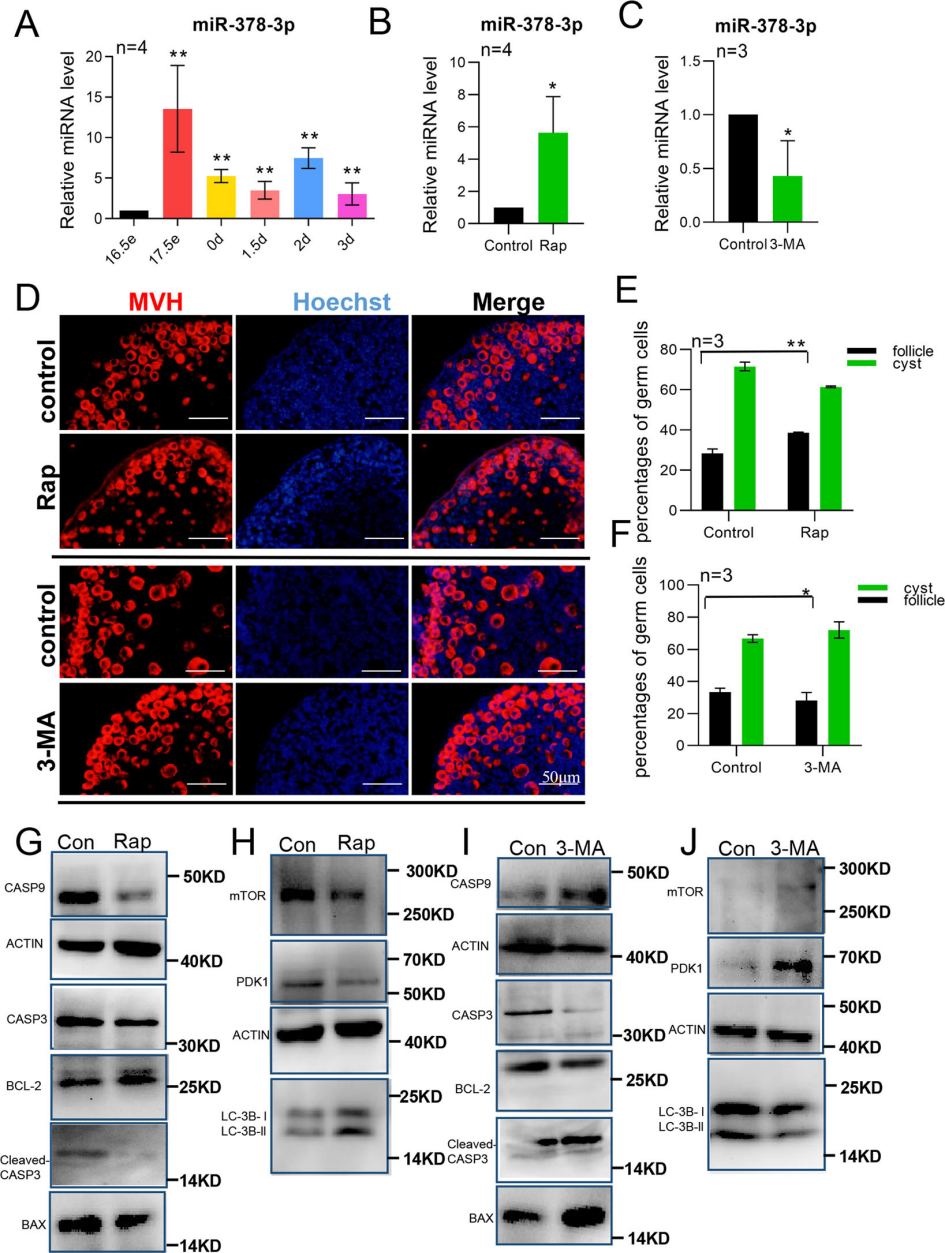
MiR-378-3p regulates primordial follicle assembly and autophagy. **a** Expression of miR-378-3p in mouse ovaries from 16.5 dpc to 3 dpp detected by miRNA qRT-PCR. The expression level of miR-378-3p significantly increased in the ovaries from 17.5 dpc to 3 dpp compared with that of 16.5 dpc; **b** Expression of miR-378-3p significantly increased after rapamycin treatment; **c** Expression of miR-378-3p significantly decreased after 3-methyladenine treatment; **d** Immunofluorescence staining of in vitro cultured mouse ovaries with rapamycin and 3-MA, respectively. Germ cells were stained red with anti-Mvh antibody, nuclei were stained blue with Hochest 33342; **e** Rapamycin increased the percentage of primordial follicle; **f** 3-MA decreased the percentage of primordial follicle; **g** Western blotting of apoptosis-related proteins CASP9, CASP3, BAX and BCL-2 after rapamycin treatment; **h** Western blotting of autophagy-related proteins PDK1, mTOR and LC3B after rapamycin treatment; **i** Western blotting of apoptosis-related proteins CASP9, CASP3, BAX, and BCL-2 after 3-MA treatment; **j** Western blotting of autophagy-related proteins PDK1, mTOR, and LC3B after 3-MA treatment.

In mouse, at 17.5 dpc germ cell cysts break down and primordial follicle assembly begin. miRNA qRT-PCR was performed to evaluate the expression of miR-378-3p during these processes. The results showed that the expression of miR-378-3p significantly increased in the ovaries from 17.5 dpc up to 3 dpp compared with that of 16.5 dpc (Fig. 1a), which suggested a role of miR-378-3p in germ cell cyst break down and primordial follicle assembly. Since autophagy is reported to be involved in primordial follicle assembly^12,14^, we cultured newborn mouse ovaries in vitro for 3 days in the presence of rapamycin and 3-methyladenine, two well-known activator and inhibitor of autophagy, respectively. The results showed a ∼5-fold increased expression of miR-378-3p in the presence of rapamycin (Fig. 1b) and a ∼2-fold decreased expression in the presence of 3-MA (Fig. 1c). At the same time, rapamycin promoted a significant increase of the percentage of germ cells in primordial follicles, while 3-MA had an opposite effect (Fig. 1d–f). These results suggested that autophagy may promote the process of germ cell cyst break down and primordial follicle assembly. In the presence of rapamycin, together with a modulation of the autophagic markers mTOR, PDK1 and LC-3B, we observed a decrease of the apoptotic protein CASP9, cleaved CASP3, and BAX (Figs. 1g, h and S2). In the presence of 3-MA, the effects on these proteins were the reverse (Figs. 1i, j and S2).

**Fig. 2:**
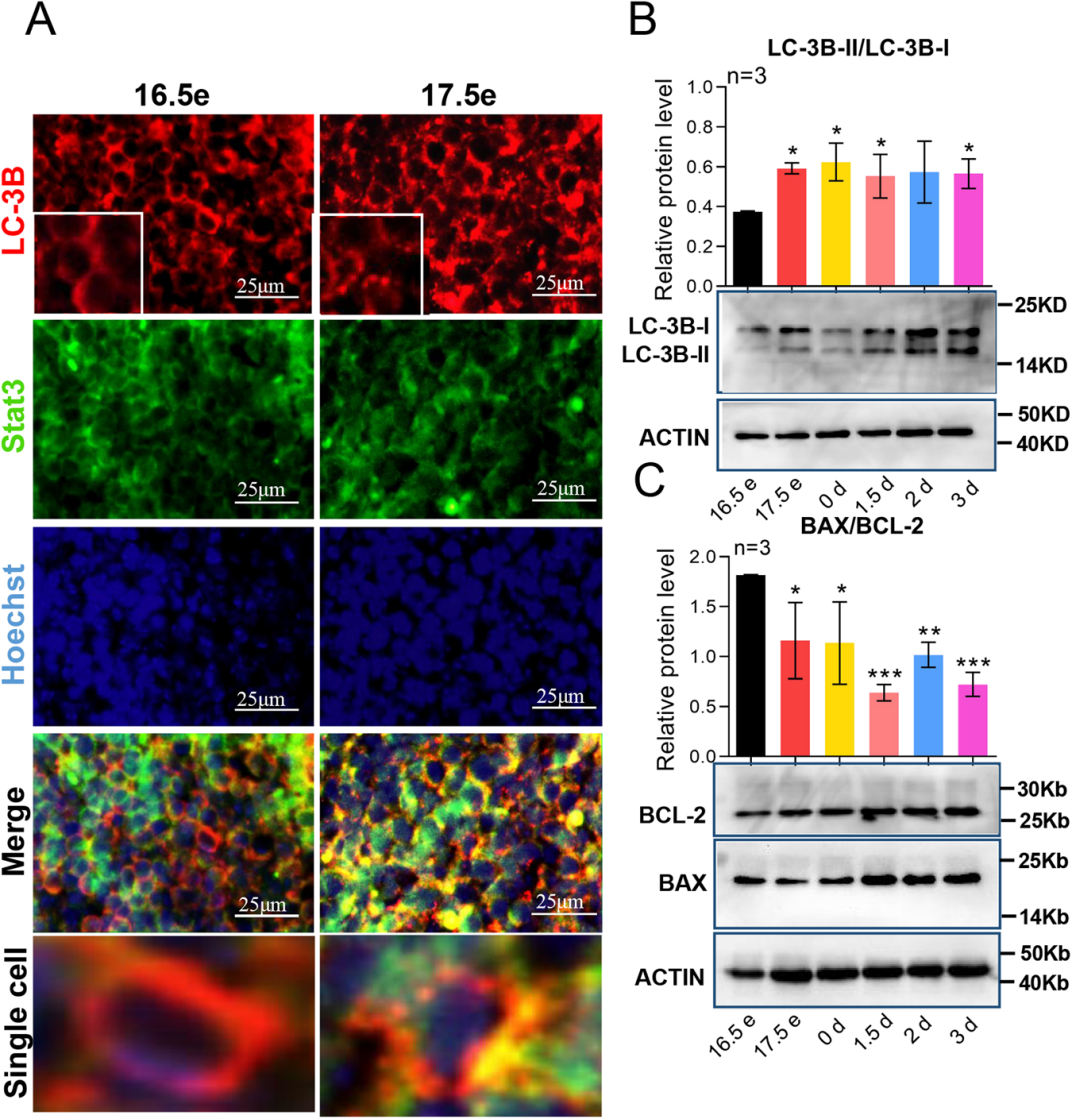
Autophagy and apoptosis were both involved in the normal process of primordial follicle assembly from 17.6 dpc to 3 dpp. **a** Comparison of autophagy of 16.5 dpc ovary and 17.5 dpc ovary by immunofluorescence staining. Germ cells were stained green with anti-Stat3 antibody, nuclei were stained blue with Hochest 33342. Punctuate structures of LC3B were stained red with anti-LC3B. In 17.5 dpc ovary more punctuate LC3B red signals were detected than that in 16.5 dpc ovary; **b** Western blot of LC3B showed the increased LC3B-II/LC3B-I after 17.5 dpc; **c** Western blot of BAX and BCL-2 showed decreased BAX/BCL-2 after 17.5 dpc.

To confirm the involvement of autophagy in primordial follicle assembly, IF staining was performed, and it was found that 17.5 dpc mice ovaries had more LC3B dots (an indication of autophagy degree) compared to those of 16.5 dpc, and by co-staining with the germ cell marker Stat3, it was clear that the dots localized in the germ cells (Fig. 2a), which meant that germ cells underwent autophagy during the process. In line with the IF analysis, western blotting for LC3B showed a significant increased expression of LC3BII/LC3B-I (Fig. 2b) and a decreased ratio of the apoptotic index BAX/BCL-2 (Fig. 2c), suggesting a decreased apoptosis accompanied with increased autophagy during germ cell cyst break down and primordial follicle assembly starting at 17.5 dpc.

### Starvation induces initially autophagy then apoptosis in newborn mouse ovaries

**Fig. 3:**
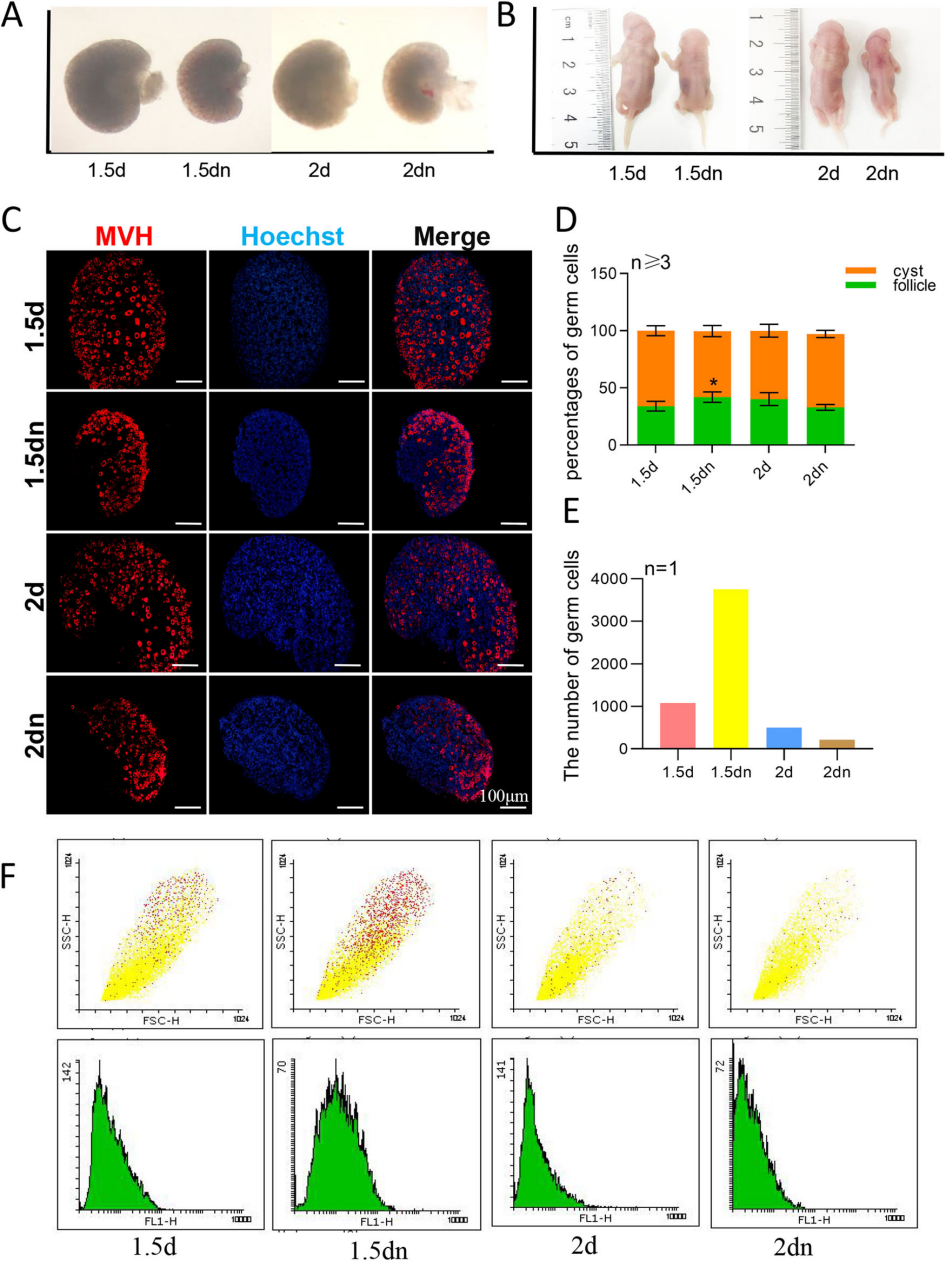
Starvation induced autophagy then apoptosis in the newborn ovaries. **a** Comparison of the size of the ovary after 1.5 days’ starvation and 2 days’ starvation; **b** Comparison of the size of the body after 1.5 days’ starvation and 2 days’ starvation; **c** Immunofluorescence staining of ovaries from 1.5 days’ starvation mice and 2 days’ starvation mice. Germ cells were stained red with anti-Mvh antibody, nuclei were stained blue with Hochest 33342; **d** Percentage of germ cells in Cyst and in primordial follicle after 1.5 days’ starvation and 2 days’ starvation; **e**, **f** Percentage of germ cells in the whole ovary after 1.5 days’ starvation and 2 days’ starvation analyzed by flow cytometry.

**Fig. 4:**
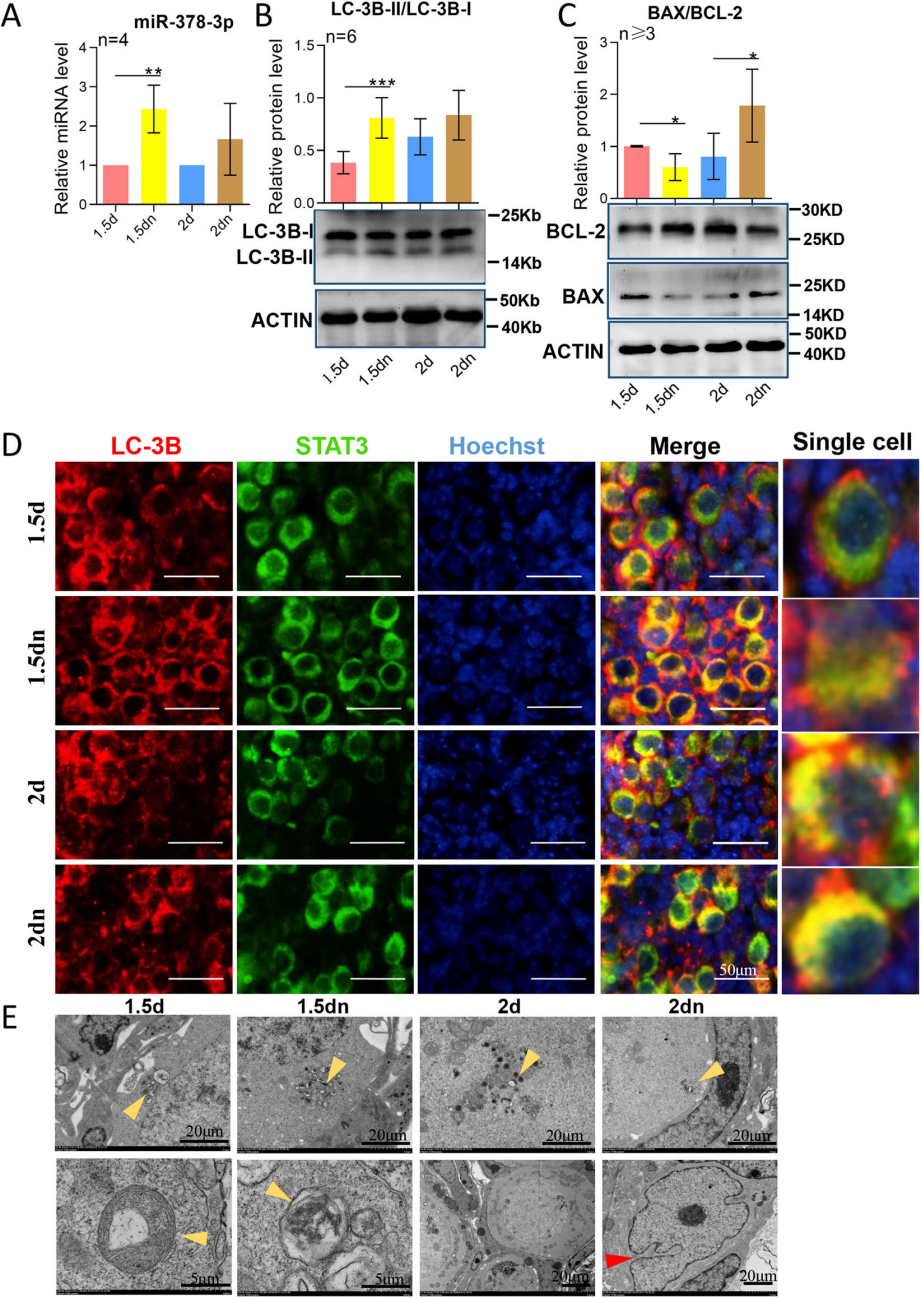
MiR-378-3p participated in primordial follicle assembly of starved mice. **a** The expression of ovarian miR-378-3p after 1.5 days’ starvation and 2 days’ starvation by miRNA qRT-PCR; **b** The protein expression of LC3BII/LC3B-I after 1.5 days’ starvation and 2 days’ starvation by western blotting; **c** The changes of apoptotic index BAX/BCL-2 after 1.5 days’ starvation and 2 days’ starvation by western blotting; **d** Comparison of autophagy level after 1.5 days’ starvation and 2 days’ starvation by immunofluorescence staining. Germ cells were stained green with anti-Stat3 antibody, nuclei were stained blue with Hochest 33342. Punctuate structures of LC3B were stained red with anti-LC3B; **e** TEM (transmission electron microscope) images of autophagosomes (yellow arrowheads) and abnormal germ cell morphology (red arrowhead).

To further confirm the roles of autophagy and apoptosis in germ cell cyst break down and primordial follicle assembly, newborn mice received no milk. Both 1.5 days’ starvation and 2 days’ starvation decreased the size of the ovary and of the body (Fig. 3a, b). It was also found that the percentage of germ cells within follicles increased after 1.5 days’ starvation, but not after 2 days’ starvation (Fig. 3c, d). The total number of germ cell also increased under 1.5 days’ starvation and decreased under 2 days’ starvation by flow cytometry (Fig. 3e, f). Moreover, increased LC3BII/LC3B-I, decreased BAX/BCL-2 and upregulated expression of miR-378-3p were found under the condition of 1.5 days’ starvation (Fig. 4a–c), consistent with the results obtained with rapamycin treatment. Increased LC3B dots (Fig. 4d) and autophagosomes (yellow arrowheads in Fig. 4e) were also found in the germ cells of newborn mice ovaries after 1.5 days of starvation. If starvation continued for 2 days, we observed that there was no significant difference in LC3B-II/LC3B-I (Fig. 4b), the apoptotic ratio of BAX/BCL-2 significantly increased (Fig. 4c) and the number of autophagosomes decreased, accompanied by abnormal germ cell morphology (red arrowhead in Fig. 4e) under TEM.

### MiR-378-3p directly targets the 3′UTR of PDK1 and Caspase9

**Fig. 5:**
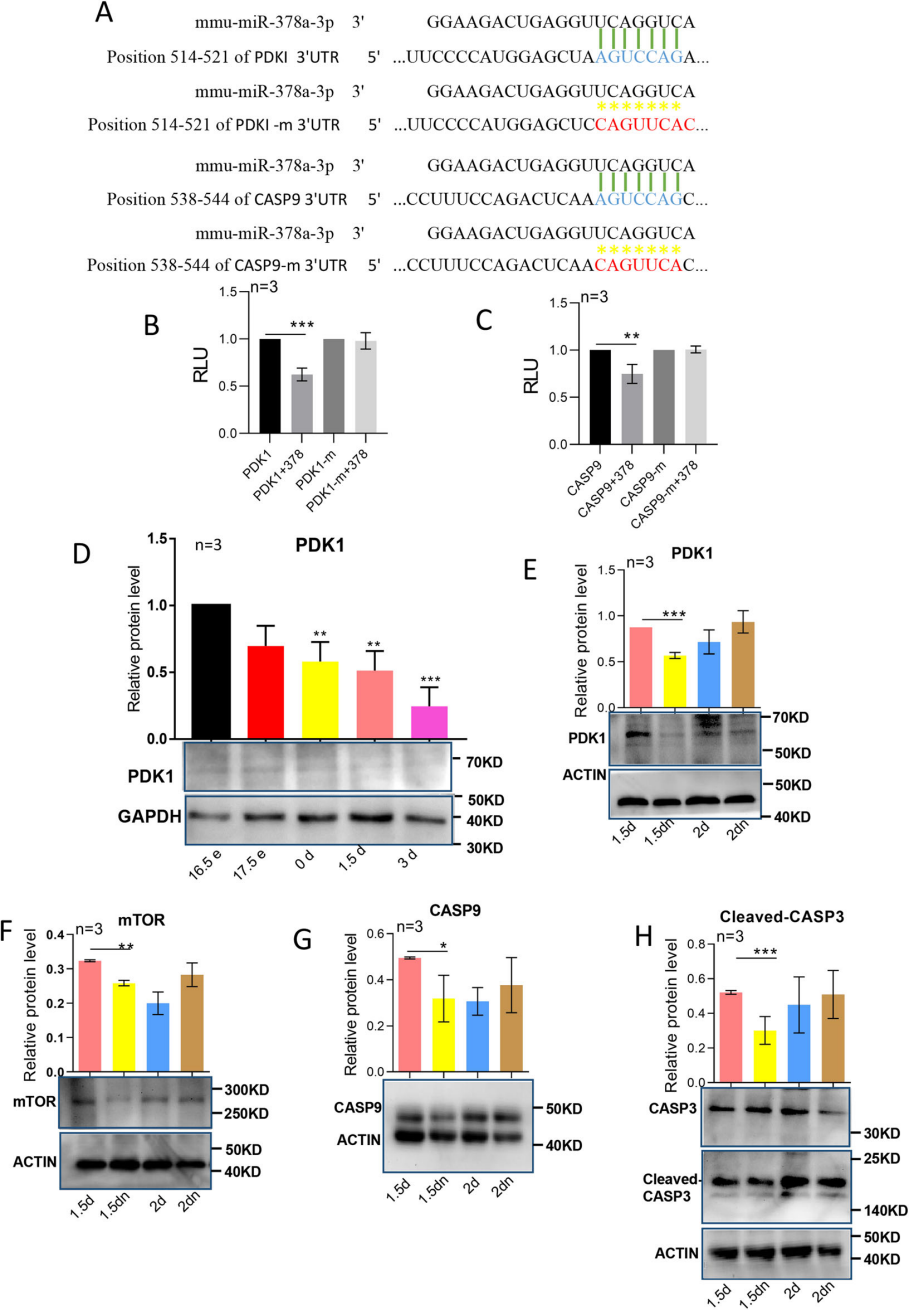
MiR-378-3p directly targeted the 3′UTR of PDK1 and Caspase9. **a** The binding position of miR-378 in target genes (PDK1 and Caspase9) and corresponding mutated sequences; **b**, **c** RLU (relative light unit) detected using the Dual Luciferase Assay System; **d** The protein expression of target PDK1 from 16.5 dpc to 3 dpp; **e**, **f** The protein expression of target PDK1 and downstream mTOR after 1.5 days’ starvation and 2 days’ starvation by western blotting; **g**, **h** The protein expression of target CASP9 and downstream CASP3 after 1.5 days’ starvation and 2 days’ starvation by western blotting.

To detect the direct targets of miR-378-3p during this process, the targets of miR-378-3p were scanned using the TargetScan software (http://www.targetscan.org/vert_72/). Among the predicted target genes, PDK1, an autophagy-related gene and Caspase9, an apoptosis-related gene caught our attention. Therefore the 200 bp 3′UTR of PDK1 and Caspase9 including the predicted miR-378-3p binding sites together with the corresponding mutant specific binding sites (Fig. 5a) were cloned into pmirGLO, a dual-luciferase miRNA target expression vector. 24 h after transfection into 293 cell line, luciferase analysis showed that with wild type 3′UTR of targets, miR-378-3p decreased luciferase activities, while with the mutant 3′UTR of targets, miR-378-3p could not decrease the luciferase activities (Fig. 5b, c).

Since we found that there was an upregulation of miR-378-3p expression from 16.5 dpc to 3 dpp and after 1.5 days of starvation, we decided to analyze its targets gene in the corresponding conditions. The PDK1 protein decreased during primordial follicle assembly (Fig. 5d), however, the total CASP9 protein had no significant decreases (Fig. S3A). After 1.5 days of starvation we observed a downregulation of autophagy inhibitory proteins PDK1 and mTOR, as well as the apoptotic proteins CASP9 and 3 (Fig. 5e–h). If starvation continued for 2 days, increased autophagy and decreased apoptosis did not continue. It showed the reverse expression trend in 2 days starvation ovaries with that of 1.5 days starvation ovaries though no significant differences were detected (Fig. 5e–h).

### MiR-378-3p overexpression induces autophagy and inhibits apoptosis

**Fig. 6:**
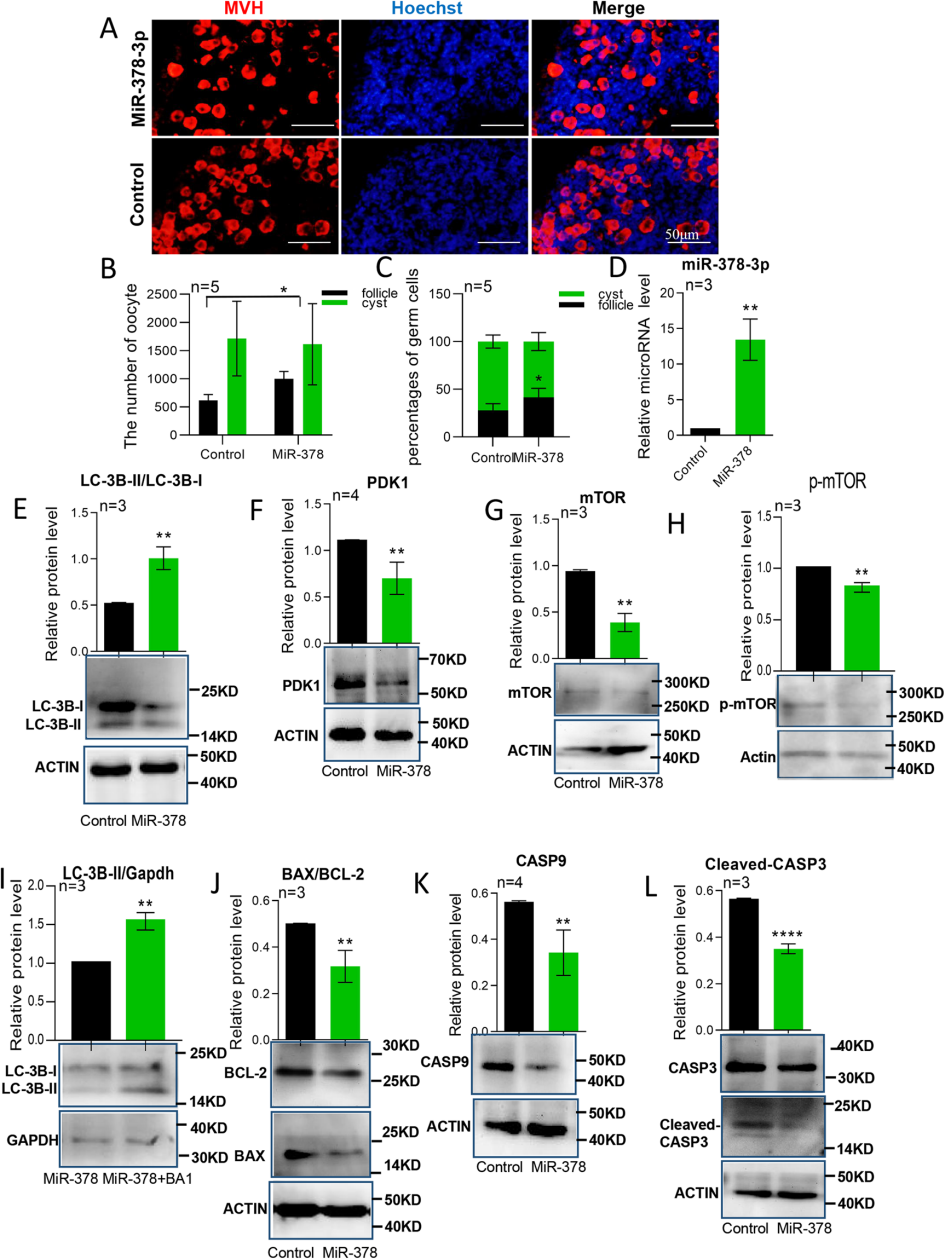
MiR-378-3p overexpression induces autophagy and inhibits apoptosis. **a** Immunofluorescence staining of in vitro cultured mouse ovaries after miR-378-3p overexpression. Germ cells were stained red with anti-Mvh antibody, nuclei were stained blue with Hochest 33342; **b** The total number of germ cells after miR-378-3p overexpression; **c** The percentage of germ cells in Cyst and primordial follicles after miR-378-3p overexpression; **d** The expression of miR-378-3p after miR-378-3p overexpression by miRNA qRT-PCR; **e**–**h** Protein expression of autophagy-related genes (LC3B, PDK1, mTOR and p-mTOR) by western blotting; **i** Autophagic flux after Bafilomycin A1 (BA1) treatment; **j**–**l** Protein expression of apoptosis-related genes (BAX, BCL-2, CASP9 and CASP3) by western blotting.

To further reveal the roles of miR-378-3p in primordial follicle assembly, we induced an overexpression of miR-378-3p in newborn mice ovaries in vitro with miR-378-3p agomir. The results showed that both the number and the percentage of primordial follicle increased (Fig. 6a–c) when miR-378-3p is over-expressed (Fig. 6d). At the same time, LC3B-II/LC3B-I levels were increased and those of PDK1, mTOR, and p-mTOR decreased (Fig. 6e–h). There was an enhanced autophagic flux (LC3B-II/GAPDH) after Bafilomycin A1 induction (Fig. 6i). And CASP9 and cleaved CASP3 were diminished, together with a decreased BAX/BCL-2 ratio (Fig. 6j–l). TUNEL staining also confirmed the decreased apoptosis (Fig. S3B, C).

### MiR-378-3p knockdown inhibits autophagy and induces apoptosis

**Fig. 7:**
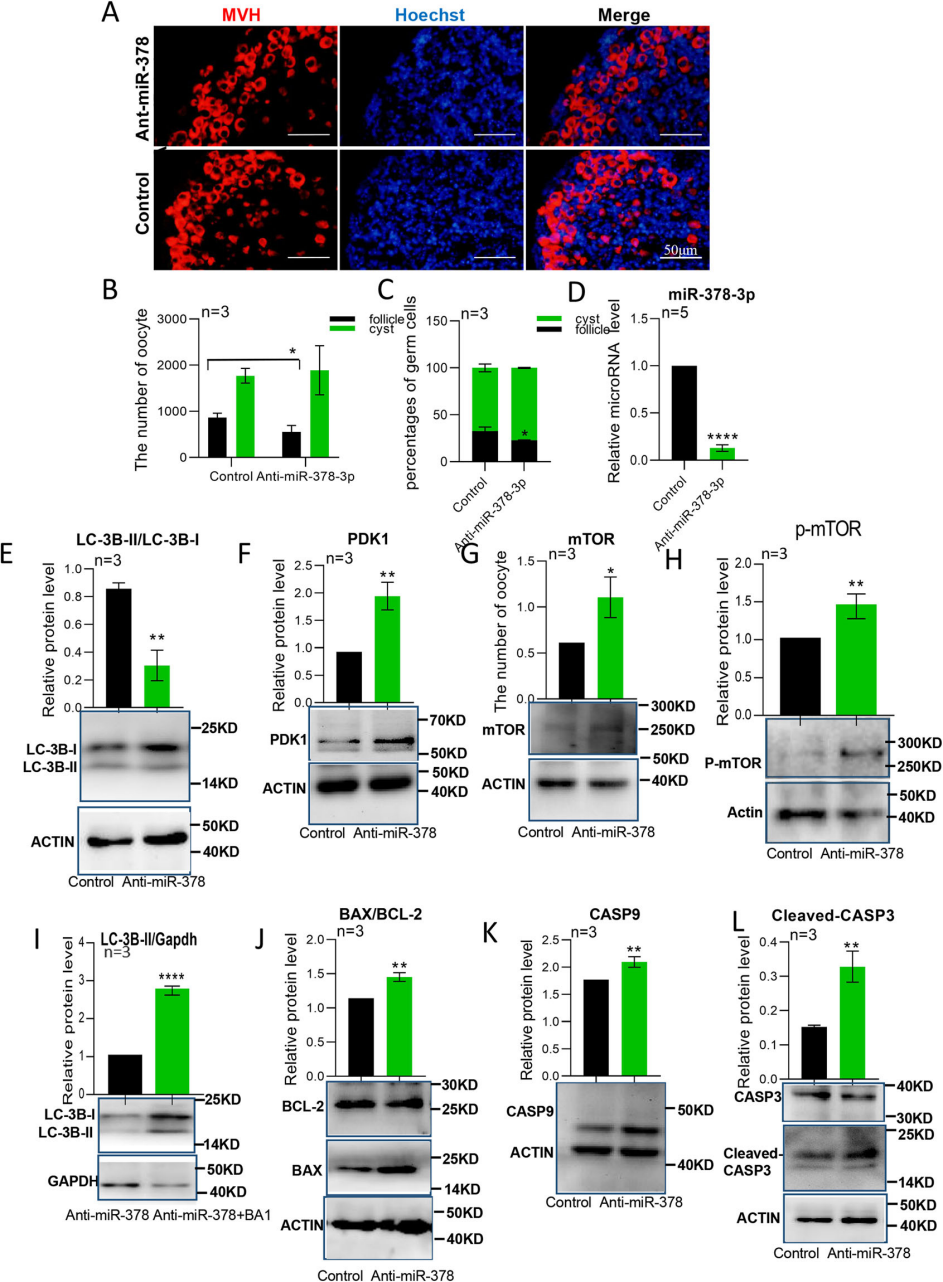
MiR-378-3p knockdown inhibited autophagy and induced apoptosis. **a** Immunofluorescence staining of in vitro cultured mouse ovaries after miR-378-3p knockdown. Germ cells were stained red with anti-Mvh antibody, nuclei were stained blue with Hochest 33342; **b** The total number of germ cells after miR-378-3p knockdown; **c** The percentage of germ cells in Cyst and primordial follicles after miR-378-3p knockdown; **d** The expression of miR-378-3p after miR-378-3p knockdown by miRNA qRT-PCR; **e**–**h** Protein expression of autophagy-related genes (LC3B, PDK1,mTOR and p-mTOR) by western blotting; **i** Autophagic flux after Bafilomycin A1 (BA1) treatment; **j**–**l** Protein expression of apoptosis-related genes (BAX, BCL-2, CASP9, and CASP3) by western blotting.

We also performed knockdown experiments in the ovaries of newborn mice using miR-378-3p antiagomir. When miR-378-3p was downregulated, the number and the percentage of primordial follicle decreased (Fig. 7a–d). As expected we observed that LC3B-II/LC3B-I levels were decreased and those of PDK1, mTOR, and p-mTOR increased (Fig. 7e–h). The autophagic flux (LC3B-II/GAPDH) increased after Bafilomycin A1 induction (Fig. 7i). And CASP9 and cleaved CASP3 were increased, as well as the BAX/BCL-2 ratio (Fig. 7j–l). TUNEL staining confirmed significantly increased apoptosis, and the apoptosis signals were mainly found in germ cells (Fig. S3B, C). To verify that the apoptosis induced by miR-378-3p inhibition was solely through Caspase9, the apoptosis-related proteins in the anti-378 treated ovaries after suppressing the expression of Caspase9 was checked by western blotting. The results showed that Caspase9 siRNA decreased the expression of CASP9 and the ratio of BAX/BCL-2 in anti-378 treated ovarian cells (Fig. S3D).

## Discussion

As it is known, two-thirds of germ cells are lost during the process of primordial follicle assembly of most mammals^8,14^. Over the last two decades, the reasons for the loss of the germ cells were extensively studied and apoptosis and autophagy are considered to be the two privileged mechanisms.

Autophagy plays an important role not only in primordial follicle assembly of mammals but also in the oogenesis in *Drosophila*^27^. In our previous study, we found that the percentage of primordial follicle decreased and apoptotic index increased in neonatal mice starved between 1.5 dpc up to 3 dpc^24^, showing opposite results with the study by Watanabe and Kimura^28^. In their study, the authors reported that autophagy in newborn mice ovarian cells, activated by non-suckling 36 h starvation, could increase the number of primordial follicle. These different results might reflect the use of different mouse strain or the different treatment time, relative to the peak of primordial follicle assembly^28^. Considering the function of autophagy on degradation and recycle of cytosol and organelles, excessive activation of autophagy may cause cell death by induction of caspase^29,30^. To further reveal the underlying mechanism, we starved the mice from birth to the limit time (2 days) of survival to research the role of autophagy and apoptosis in primordial follicle assembly and also to outline the role of miR-378 during such process. Our results showed the promoted autophagy and inhibited apoptosis during germ cell cyst break down and primordial follicle assembly, which suggested the antagonism relationship between autophagy and apoptosis (Fig. 2a–c). In our study, during primordial follicle assembly (17.5 dpc–3 dpp), starvation for 1.5 days and miR-378 overexpression caused increased autophagy and decreased apoptosis (Fig. 3). But starvation for 2 days, increased apoptosis (Fig. 3). We found the nutrition deprivation for 2 days is the life limit point, since longer nutrition deprivation leads to mice death. After 1.5 days starvation treatment, the nutrition and the energy were unable to maintain cell survival, or excessive induction of autophagy injured the cytosol and organelles leading to the loss of the cellular functions and cell morphology. Actually, autophagy and apoptosis are not existing independently; they can crosstalk with each other in the cells by the direct binding of Beclin1, an important mediator in autophagy signaling pathway, which contains BH3 domain, and BCL-2 family proteins with BH3 binding grooves^31,32^. Embryonic fibroblasts from Bax/Bak double knockout mice still underwent a non-apoptotic death after death stimulation, though they were resistant to apoptosis. And it was proved that the non-apoptotic death was associated with autophagosomes/autolysosomes, due to the suppression of the death by inhibitors of autophagy^33^.

MiRNA, as one of the epigenetic regulator, plays several roles in animal reproductive development and attracted more and more attention. Pan and his colleagues reported that the expression of miR-378 in cumulus cell of pig ovary was time-specific between GV and MII stage^21^. In this study, we found as well a time-specific expression of miR-378-3p in mouse ovary during primordial follicle assembly. During this process, the expression of miR-378-3p increased (Fig. 1a); while if miR-378-3p was downregulated, the process of cyst breakdown and primordial follicle assembly was inhibited leading to a lower percentage of primordial follicles (Fig. 7b, c). However, if miR-378-3p was upregulated, the process of cyst breakdown and primordial follicle assembly was promoted, leading to the higher percentage of primordial follicle (Fig. 6b, c). These results showed a direct role of miR-378-3p in the size of primordial follicle pool. We also found 2 targets of miR-378-3p, PDK1, and Caspase9 (Figs. 5 and S1). PDK1 is the negative regulator of autophagy, which can inhibit autophagy at the level of the mTOR pathway^34,35^. The relationship between miR-378 and it’s target PDK1 provided the evidence that miR-378 could promote autophagy. Meanwhile, miR-378-3p were increased after the autophagy was induced by rapamycin, and the levels of miR-378-3p were decreased after the autophagy was inhibited by 3-MA (Fig. 1b, c). These results seemed that autophagy was able to increase the expression of miR-378-3p. Whether there is a feedback loop needs to be further confirmed. CASP9 is an initiator caspase activated by cytochrome c released from mitochondria (mitochondrial-mediated intrinsic apoptotic pathway)^36,37^. During primordial follicle assembly, increased miR-378-3p degraded PDK1 by post-transcriptional regulation mechanism (Fig. 6f), then downregulated mTOR and activated pro-survival autophagy. At the same time, increased miR-378-3p targeted Caspase9 and inhibited apoptosis. The same situation occurred in the ovaries after 1.5 days starvation with increased miR-378-3p expression. But during 2 days starvation, the expression of miR-378-3p had no significant difference with the control, leading to increased apoptosis (Fig. 4c). So we concluded that miR-378-3p regulates the size of primordial follicle pool by upregulating autophagy and downregulating apoptosis to avoid over-loss of germ cells. MiR-378-3p, might be a target on establishment of primordial follicle pool, regulation the size of primordial follicle pool, and enhancement of reproductive potential of female mammals.

## Supplementary information

Figure S1

Figure S2

Figure S3

supplementary figure legends
