# Supplementary figures and images for: miR-378-3p maintains the size of mouse primordial follicle pool by regulating cell autophagy and apoptosis
Source: Cell Death Dis. 2020 Sep 10;11(9):737. doi: 10.1038/s41419-020-02965-1 (PMC7483766; doi:10.1038/s41419-020-02965-1)

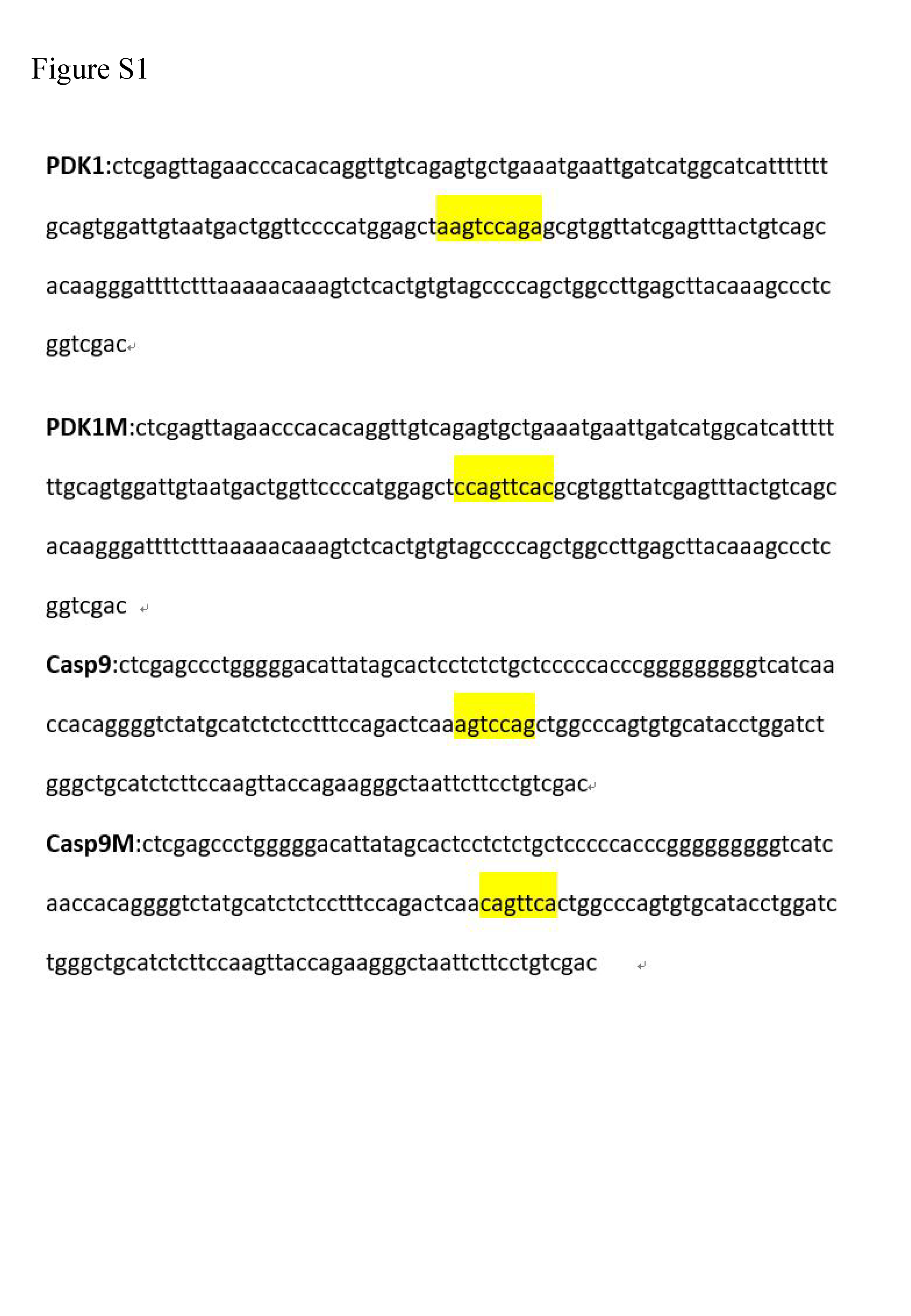

Supplement: Supplementary file 1 — Figure S1 [file 41419_2020_2965_MOESM1_ESM.tif]

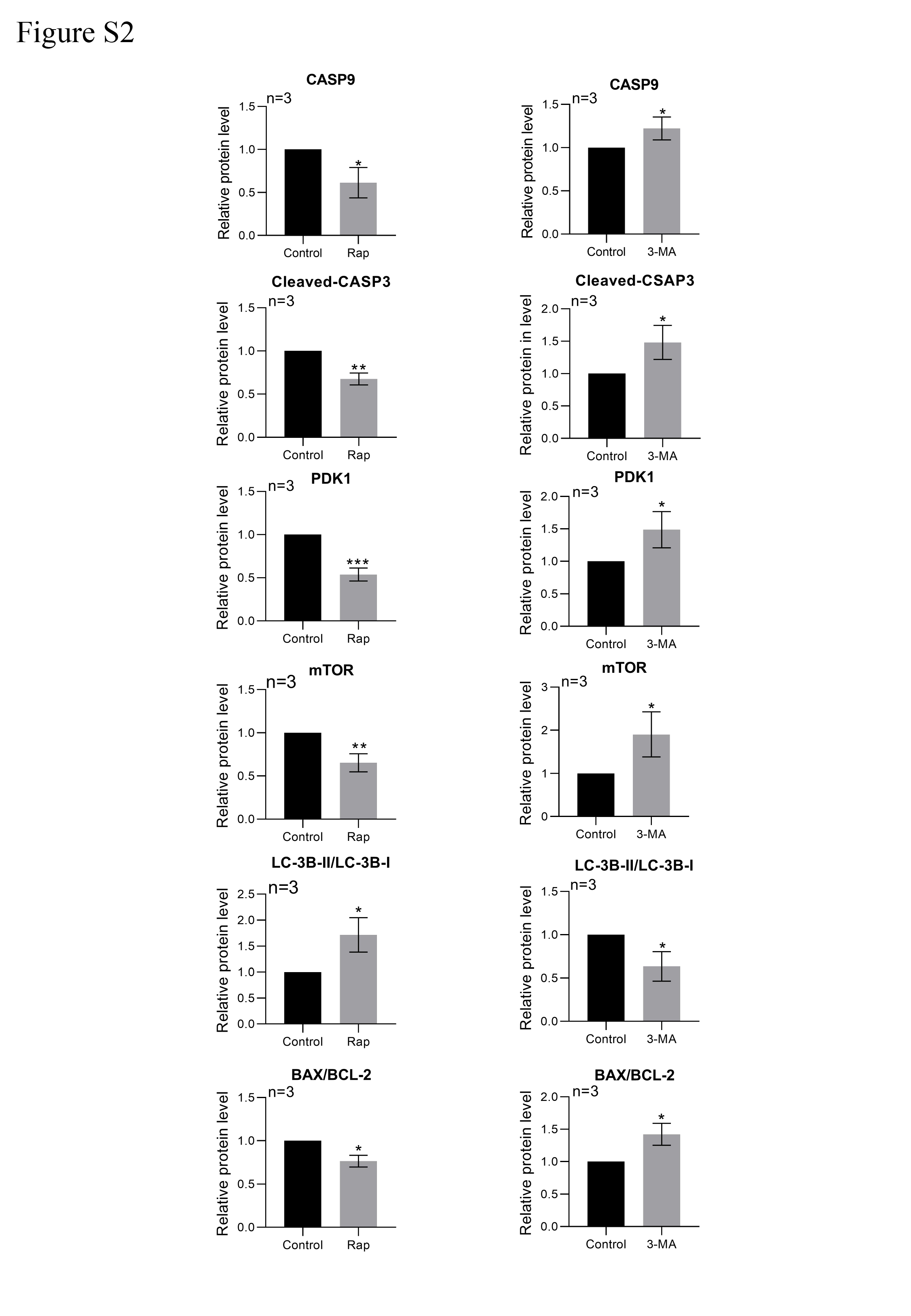

Supplement: Supplementary file 2 — Figure S2 [file 41419_2020_2965_MOESM2_ESM.tif]

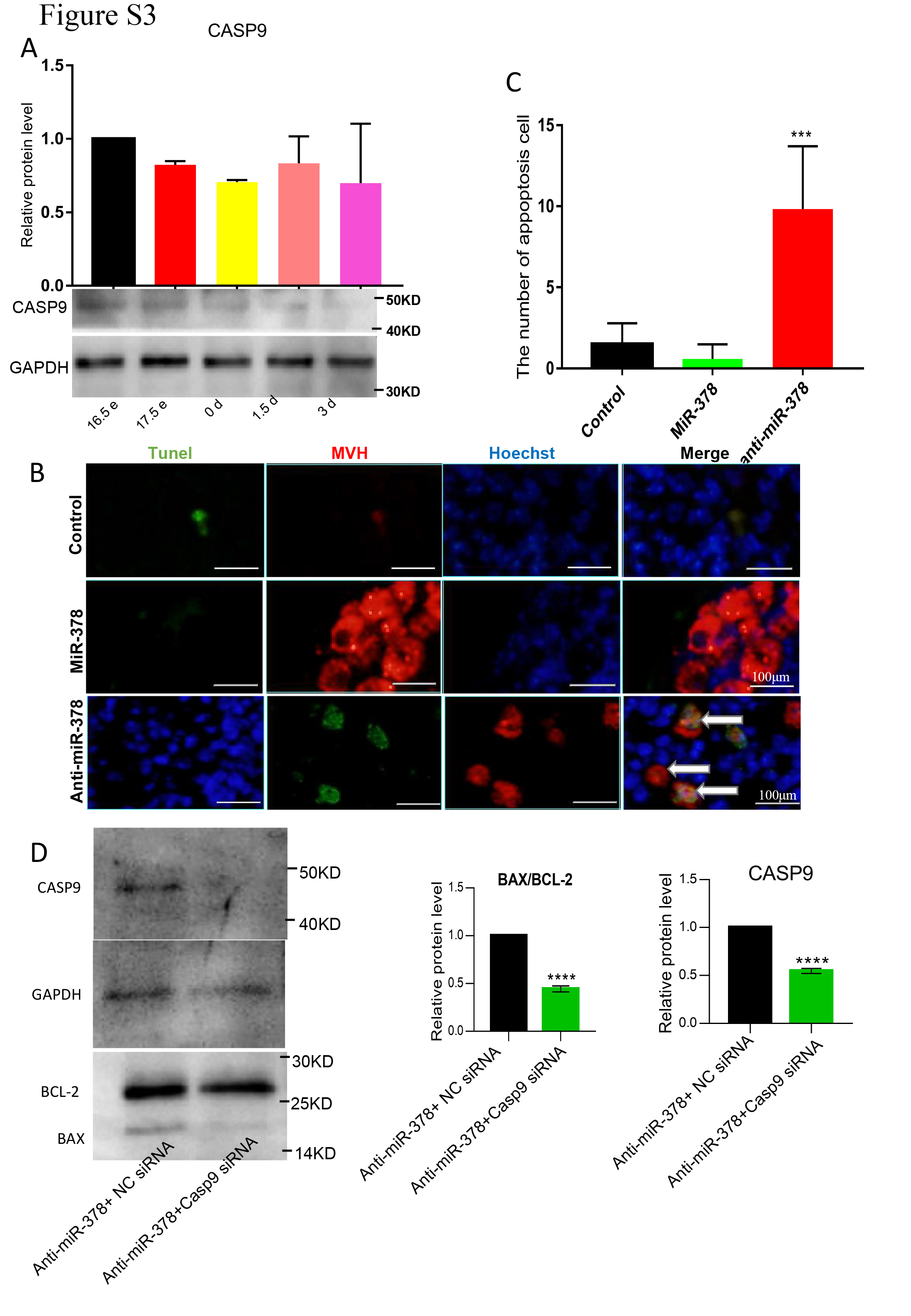

Supplement: Supplementary file 3 — Figure S3 [file 41419_2020_2965_MOESM3_ESM.tif]
