# Supplementary material for: miR-378-3p maintains the size of mouse primordial follicle pool by regulating cell autophagy and apoptosis
Source: Cell Death Dis. 2020 Sep 10;11(9):737. doi: 10.1038/s41419-020-02965-1 (PMC7483766; doi:10.1038/s41419-020-02965-1)
Supplement: Supplementary file 4 — supplementary figure legends [file 41419_2020_2965_MOESM4_ESM.docx]

Figure S1: The detailed 3’UTR sequences cloned into pmirGLO Dual-Luciferase miRNA Target Expression Vector.

Figure S2: Quantitative analysis of proteins in Fig. 1G-J.

Figure S3: (A) Protein expression level of CASP9 from 16.5 dpc to 3 dpp; (B and C) TUNEL staining after miR-378-3p over-expression and miR-378-3p knock down. (D) Protein expression of CASP9 and the ratio of BAX/BCL-2 in anti-378 treated ovarian cells after Caspase9 gene silencing.
